# SOCIAL NETWORK SIZE AND FREQUENCY OF ELECTRONIC COMMUNICATION: THE RELATIONSHIP TO INTERPERSONAL STRESS

**DOI:** 10.1093/geroni/igz038.618

**Published:** 2019-11-08

**Authors:** Xin Yao Lin, Margie E Lachman

**Affiliations:** Brandeis University, Waltham, Massachusetts, United States

## Abstract

Social relationships are beneficial for psychological wellbeing, but they are also associated with interpersonal stress. With the growing usage of multiple forms of electronic communications (EC) including phone calls, text messages, video chat, and internet among adults of all ages, it was of interest to explore the relationship between social network size (SNS), in-person communication (PC), and EC, and whether the relationship between SNS and frequency of communication is associated with interpersonal stress. A daily diary study was conducted over seven days for 142 participants ages 22 to 94. SNS was assessed with the social convoy model. Frequency of PC and EC, along with interpersonal stress, were assessed daily. As expected, multiple regression analysis results showed that older adults had smaller SNS and less frequent technology communication (text messages, video chat, internet) compared to younger adults. With regard to effects on interpersonal stress, there were no main effects for frequency of PC, EC, or SNS. However, the frequency of EC moderated the relationship between SNS and interpersonal stress, controlling for amount of PC. Among those with a smaller SNS, having more frequent EC was associated with less interpersonal stress compared to those with less frequent EC. For those with a larger SNS, having more frequent EC was associated with more interpersonal stress compared to those with less EC, but PC was not related to interpersonal stress. The discussion will consider implications of the findings for developing interventions to minimize stress from interpersonal communications, especially those that involve EC.

